# Anti-regulatory T cells are natural regulatory effector T cells

**DOI:** 10.15698/cst2019.10.199

**Published:** 2019-10-08

**Authors:** Niels Ødum

**Affiliations:** 1 The LEO Foundation Skin Immunology Research Center, Department of Immunology and Microbiology, University of Copenhagen, Blegdamsvej 3B, DK-2200 Copenhagen, Denmark.

**Keywords:** anti-regulatory T cells, PD-L1, Treg, IDO, inflammation, immunotherapy

It is well established that the immune system uses regulatory immune-suppressive cells to inhibit and terminate immune reactions and maintain immune balance. In the last decade, Andersen and colleagues have discovered that regulatory cells also can have effector capabilities that counteract the many immune-suppressive feedback mechanisms that regulatory cells mediate. These authors have described pro-inflammatory antigen-specific T cells that react towards immune-suppressive cells [1,2]. Indeed, because of their reactive ability against regulatory immune cells, these effector T cells have been designated as anti-regulatory T cells or anti-Tregs [3]. Anti-Tregs recognize proteins that regulatory cells express, including PD-L1 [4–7].

Spontaneous CD8+ and CD4+ T-cell reactivity against PD-L1 has been described in patients with cancer and in healthy individuals. Naturally occurring PD-L1–specific T cells can recognize PD-L1–expressing immune cells and malignant cells [8]. Activation of PD-L1–specific T cells has been described as modulating adaptive immune reactions directly and indirectly. The addition of PD-L1–specific T cells to cultured peripheral blood mononuclear cells (PBMCs) one week after viral antigen stimulation results in an immense increase in virus-specific T cells. Likewise, the co-stimulation of PD-L1 epitopes with viral epitopes results in expansion of virus-specific T cells. Thus, activation of PD-L1–specific T cells enhances the effector phase of an ongoing immune response.

In the current issue of *Cell Stress*, Andersen and colleagues further characterize the natural function of PD-L1–specific T cells, showing a direct link between inflammation and expansion of this cell population [9]. PD-L1 is expressed even in very potent antigen-presenting cells early during the inflammatory process. This expression occurs because of induction by both type I and II interferons (IFNs), which are present at the inflammation site. PD-L1 thus plays a central role in the counter-regulation of immune responses.

Andersen and colleagues also show that circulating PD-L1–specific T cells expand in response to pro-inflammatory mediators, such as IFN-γ and interleukin-2, in the absence of antigen-specific stimulation. PD-L1–specific T cells therefore expand as a first response to inflammation and can function as helper cells at the inflammation site, where they also can aid in the response to infected cells. Further evidence for these roles is the increased susceptibility of target cells to PD-L1–specific T-cell recognition in the presence of IFN-γ [4]. In their current work in *Cell Stress*, Andersen *et al.* provide further support for the natural regulatory role of PD-L1–specific anti-Tregs, showing that addition of inflammation-induced PD-L1–specific T cells to unstimulated PBMC cultures indeed influences Treg numbers [9].

PD-L1 is not the only target that regulatory immune cells express and that anti-Tregs can recognize. The metabolic enzymes indoleamine-pyrrole 2,3-dioxygenase (IDO) and arginase are likewise identified as antigens that pro-inflammatory T cells recognize in individuals with and without cancer [10–13]. Arginase is of particular interest because it is not induced by Th1 cytokines, as are IDO and PD-L1, but rather by Th2 cytokines. Thus, pro-inflammatory arginase-specific T cells might represent yet another kind of anti-Tregs with a natural function in settings other than those involving Th1-induced IDO- or PD-L1–specific anti-Tregs. In anti-cancer immunotherapy, vaccination-induced activation of anti-Tregs is emerging as a novel way to target the immune-suppressive tumor microenvironment [14,15]. An understanding of the different types of anti-Tregs may be crucial for the development of such immune-modulatory vaccines. Activation of arginase-specific T cells by vaccination should cause T-cell infiltration at the tumor site, even in tumors with an “excluded” phenotype, without infiltrating T cells; arginase-expressing cells, such as tumor-associated macrophages (TAMs), have been described as excluding CD8(+) T cells from the tumor parenchyma [16]. Because Th1 inflammation signals, such as IFN-γ, spontaneously lead to expansion of PD-L1–specific T cells, arginase-based vaccination could potentially act synergistically with PD-L1–based vaccines [17]. In this scenario, an arginase vaccination should induce Th1 inflammation at tumor sites where TAMs otherwise prevent lymphocyte infiltration. In turn, this effect would induce PD-L1 activation, which could enable further targeting by PD-L1–specific anti-Tregs. Thus, a combination of epitopes from different anti-Treg target antigens could yield additive effects in an immune-modulatory vaccination approach.

In conclusion, anti-Tregs can directly eliminate regulatory immune cells and indirectly augment the effector function of other immune cells through direct suppression of immune-suppressive cells and release of pro-inflammatory cytokines. The results reported by Andersen *et al.* in the current issue of *Cell Stress* [9] emphasize the potentially important immune-regulatory role of PD-L1–specific effector T cells that are activated in response to inflammatory mediators, which are part of any inflammatory reaction.
